# *Lawsonia intracellularis* T3SS effector LI0758, an Rce1 ortholog, activates MAPK and NF-κB signaling in mammalian cells

**DOI:** 10.1186/s13567-025-01461-8

**Published:** 2025-02-04

**Authors:** Yuanxiu Zhong, Yiyun Duan, Fenju Lai, Jinhua Zhang, Yimin Dai

**Affiliations:** 1 School of Animal Science and Technology, Jiangxi Agricultural University, Nanchang, 330045 China; 2 School of Biological Science and Engineering, Jiangxi Agricultural University, Nanchang, 330045 China

**Keywords:** *L. intracellularis*, type III secretion system, MAPK, NF-κB, prenylation, Rce1

## Abstract

*Lawsonia intracellularis*, a Gram-negative obligate intracellular bacterium causing porcine proliferative enteropathy, possesses a type III secretion system (T3SS), yet only a handful of its substrates have been experimentally characterized. In this study, we identify that LI0758 can be secreted by the Yersinia T3SS, which suppresses yeast growth and activates mitogen-activated protein kinase (MAPK) and nuclear factor-kappa B (NF-κB) signaling pathway in mammalian cells. Bioinformatics analyses indicate that LI0758 is an ortholog of Rce1, a eukaryotic CAAX protein endoprotease, sharing a similar subcellular localization on the endoplasmic reticulum (ER). While displaying unique activity in the yeast a-factor reporter system, LI0758 restores Ras2 localization in Rce1Δ mutant strains, implying functional similarity. Our findings underscore LI0758’s pivotal role in activating MAPK pathways and suggest its potential to modulate the localization and function of host CAAX proteins. Further investigation holds promise for elucidating novel bacteria-host interaction mechanisms and fostering the development of innovative therapies against proliferative enteritis.

## Introduction

*Lawsonia intracellularis*, a Gram-negative, anaerobic obligate intracellular bacterium, is the sole species in the bacterial genus Lawsonia and is the etiologic agent of proliferative enteropathy (PE) [1]*. L. intracellularis* typically infects the small intestine of pigs, although occasional infections of the large intestine have been reported in pigs and other animals, such as hamsters and horses [1]. The infection presents with symptoms including proliferation, hemorrhage, necrosis, commonly known as “ileitis”, which compromises the health and productivity of farmed pigs [2]. Prophylactic measures against *L. intracellularis* infection in pigs encompass the administration of subtherapeutic levels of in-feed antibiotic growth promoters or vaccination. Although the former strategy has demonstrated efficacy in controlling *L. intracellularis*, the anticipated regulatory restrictions on subtherapeutic antibiotics in numerous countries highlight the need for alternative approaches [2]. Despite extensive research over several decades that has elucidated the infection dynamics of *L. intracellularis* both in vitro and in vivo, the underlying pathogenesis and virulence factors of this organism remain poorly understood [3].

The type III secretion system (T3SS), a well-studied mechanism, plays crucial roles in the pathogenicity of numerous Gram-negative bacterial pathogens [4]. Currently, several enteropathogenic bacteria are known to utilize T3SS for infecting their hosts and causing various infectious diseases [5]. The Ysc-Yop T3SS of Yersinia, often regarded as the archetypal T3SS, was the first to be characterized [6]. Due to the conserved structural framework of T3SS across different animal bacterial pathogens, Yersinia has been effectively used as a model system to identify novel T3SS substrates in genetically intractable strains [7]. The genes encoding the T3S apparatus have been identified in the complete genome of *L. intracellularis* strain PHE/MN1-00, and the expression of some components has been confirmed through RT-PCR and serological response during infection [8]. Given the microaerophilic obligate intracellular lifestyle of *L. intracellularis*, identifying its T3SS substrate directly presents a significant challenge [2]. Recently, our laboratory utilized the Yersinia T3SS in vitro secretion system to successfully identify two effectors of *L. intracellularis*, LI0666 and LfliC, along with two T3SS translocators, LI1158 and LI1159 [9–11].

Prenylation, defined as the covalent attachment of isoprenoid lipids to eukaryotic proteins, exerts regulatory control over the subcellular localization and functional activity of a diverse array of proteins that play pivotal roles in biological regulatory processes [12]. Most prenylated proteins are categorized as CAAX proteins, wherein the prenylation process is initiated by the ligation of either a 15-carbon farnesyl or a 20-carbon geranylgeranyl isoprenoid lipid to the cysteine (Cys) residue [12]. Subsequently, these proteins can undergo further enzymatic processing: first by RAS-converting CAAX endopeptidase 1 (RCE1), which catalyzes the removal of the -AAX sequence, and then by isoprenylcysteine carboxylmethyltransferase (ICMT), which methylates the carboxyl group of the resulting carboxyterminal isoprenoid-modified Cys residue, effectively “capping” it with a methyl group [12].

Rce1, an integral membrane protease residing in the endoplasmic reticulum (ER), was originally discovered in *Saccharomyces cerevisiae* through genetic and biochemical studies of the yeast a-factor [13]. It functions as a type II CAAX prenyl endopeptidase, collaborating with the type I CAAX-processing enzyme ZMPSTE24/Ste24p (also known as AFC1p) [13]. ZMPSTE24/Ste24p is a zinc metalloprotease with a distinct role in processing prelamin A in all eukaryotes and the a-factor specifically in yeast. Conversely, Rce1 demonstrates broader specificity, catalyzing the processing of all farnesylated and geranylgeranylated CAAX proteins, encompassing the a-factor in yeast and members of the Ras superfamily of small GTPases [14].

The MAPK (Mitogen-Activated Protein Kinase) pathway is widely acknowledged as a fundamental and prototypical downstream element of the Ras signaling cascade, serving as a pivotal intermediary in transmitting signals from Ras to evoke cellular responses [15]. These MAPK-signaling cascades constitute ubiquitous processes within eukaryotic cells and are frequently targeted by T3SS effectors of enteropathogenic bacteria to modulate the host’s innate immune response [5]. Despite the divergence in the outputs of these signaling cascades across different organisms, ranging from yeast to mammals, the signaling components of these pathways exhibit a high degree of conservation [16].

In yeast, five distinct and well-documented MAPK signaling pathways have been characterized: the pheromone response pathway (Fus3), the filamentous growth pathway (Kss1), the hyperosmotic/glycerol growth pathway (Hog1), the cell wall integrity pathway (Slt2), and the spore wall assembly pathway (Smk1) [16]. In mammalian cells, MAP kinases can be classified into three primary families: the ERKs (Extracellular-Signal-Regulated Kinases), the JNKs (Jun Amino-Terminal Kinases), and the p38 kinases [17].

Rce1 is a member of the ABI (Abortive Infection) family of putative integral membrane proteases, which lacks paralogs in eukaryotes [14]. Although prenylation modifications are not observed in prokaryotic cells and archaebacteria, numerous Rce1p orthologs have been identified across all three domains of life [18, 19]. The pair-wise identity among RCE1 orthologs varies between 14 and 27%. Despite this relatively low level of primary sequence conservation, all examined Rce1p orthologs have been found to be capable of substituting for yeast Rce1p in the maturation of the yeast a-factor mating pheromone [14, 19, 20]. These findings imply that the RCE family may possess conserved substrate specificity.

In this study, we reveal LI0758 as a T3SS effector exported by Yersinia, inhibiting yeast growth and significantly activating MAPK pathways in HEK293T cells. Bioinformatics analyses suggest LI0758 is an ortholog of Rce1, with similar localization on the ER. Although it exhibits distinct in vivo activity in the yeast a-factor reporter system, LI0758 restores Ras2 localization in Rce1Δ mutant strains, suggesting a functional resemblance. These findings highlight LI0758’s role in MAPK pathway activation and suggest it may regulate host CAAX proteins’ localization and function. Further exploration promises to uncover new bacteria-host interaction patterns and pave the way for innovative drug development against proliferative enteritis.

## Materials and methods

### Strains, cell lines, and medium

The plasmid amplification was performed using *E. coli* DH5α (TransGen Biotech, Beijing, China), which was cultivated in Luria–Bertani (LB) medium containing 100 μg mL^−1^ ampicillin at 37 ℃ with agitation or on LB agar plates. T3S-proficient *Y. pseudotuberculosis* IP2666Δ^yopHOPEMT^, *Y. enterocolitica* MRS40^ΔyopHOPEM^ and T3SS-deficient *Y. enterocolitica* MRS40^ΔyspF^ were routinely grown in brain heart infusion (BHI; TransGen Biotech) agar, and 200 µg mL^−1^ ampicillin was added to select expression vectors.

The yeast strains used in this study, along with the primers used for their construction, are listed in Tables 1 and 2. Briefly, W303-1A and BY4741 were employed for growth inhibition assays, whereas W303-1A and W303-1B served in quantitative mating assays. Furthermore, W303-1A was specifically selected for the RAS2 localization assay. To investigate CAAX protease specificity, two pairs of primers were used to generate three strains in the W303-1A background, each lacking the CAAX protease genes (RCE1 and/or STE24). Specifically, the rce1Δ strain replaces the RCE1 open reading frame with TRP1, the ste24Δ strain replaces the STE24 open reading frame with HIS3, and the rce1Δste24Δ double mutant strain replaces both the RCE1 and STE24 open reading frames with TRP1 and HIS3, respectively. All deletion strains were verified through PCR amplification of the corresponding loci using test primers. All strains were cultured at either 30 ℃ or 37 ℃ in yeast extract peptone dextrose (YPD) or synthetic complete drop-out (SC) medium.

**Table 1:** **Yeast strains used in this study**

Strain	Relevant genotype	Source
W303-1A	*MAT*a *ade*2-1 *ura*3-1 *his*3-11,15 *trp*1-1*, leu*2-3,112 *can-*100	Clontech
W303-1B	*MAT*α* ade*2-1 *ura*3-1 *his*3-11,15 *trp*1-1*, leu*2-3,112 *can-*100	Clontech
BY4741	MATa his3Δ1, leu2Δ0, met15Δ0, ura3Δ0	Clontech
W303-1A Rce1Δ	*MAT*a *ade*2-1 *ura*3-1 *his*3-11,15 *trp*1-1*, leu*2-3,112 *can-*100 RCE1::TRP1	This study
W303-1A ste24Δ	*MAT*a *ade*2-1 *ura*3-1 *his*3-11,15 *trp*1-1*, leu*2-3,112 *can-*100 ste24::HIS3	This study
W303-1A rce1Δste24Δ	MATa ade2-1 ura3-1 his3-11,15 trp1-1, leu2-3,112 can-100 RCE1::TRP1 ste24::HIS3	This study

**Table 2:** **Primers used in this study**

Primer	Sequence 5′—3′ (Restriction enzyme sites are underlined)
ScRce1-TRP1-recombinant-F	CCTTTGATGATTTTATTACCTTTATTTTAAGTTACTAAAATATCGAGATTGTACTGAGAGTGCACC
ScRce1-TRP1-recombinant-R	AAACAGTTGTCATGGAGCCTTCCTGTAATTGCTCATAAGCATGGTGTGCGGTATTTCACACCGC
ScSte24-HIS3-recombinant-F	GAAAAAAAAGGAGGAAATAGAAAACTGCAGGCCTTTATTCATGACAGAGCAGAAAGCCCT
ScSte24-HIS3-recombinant-R	CTGAATTTAACGGTACATGCTAATATGTGTACTCTATAGACTACATAAGAACACCTTTGG
rce1Δ(rce1::trp1)-TEST-F	AAACCATCTTGGCGTAGCG
rce1Δ(rce1::trp1)-TEST-R	AGACTTCCGTTTATCCCTTT
ste24Δ(ste24::his3)-TEST-F	GGCAGATGTGGAAGGTAAGA
Ste24Δ(ste24::his3)-TEST-R	ATACTTCCAATCTGTTGTGCC
pBAD24-LI0758-F	GAGGAATTCATGAAAAATCATACACCT
pBAD24-LI0758-R	GTAGTCGACACCTAAAATAGGGAAAAG
pRS416-LI0758-F	GAGGAGCGGCCGCGATGAAAAATCATACACCT
pRS416-LI0758-R	CATGACTCGAGTTAACCTAAAATAGGGAAAAG
pRS416-ScRce1-F	GAGGAGCGGCCGCGATGCTACAATTCTCAACATTTC
pRS416-ScRce1-R	CATGACTCGAGCTAAAGGGTTATTCTATAAC
pRS416-mCherry-F	AAGGAAAAAAGCGGCCGCGATGGTGAGCAAGGGCGAG
pRS416-mCherry-R	CCGCTCGAGTTACTTGTACAGCTCGTC
pRS416-mCherry-LI0758-F	CCGCTCGAGATGAAAAATCATACA
pRS416-mCherry-LI0758-R	CGGGGTACCTTAACCTAAAATAGG
pRS416-mCherry-ScRce1-F	CCGCTCGAGATGCTACAATTCTCAACA
pRS416-mCherry-ScRce1-R	CGGGGTACCTTATTCTATAACCAGGAG
pESC-LEU-EGFP-F	CCCTCGAGATGGTGAGCAAGGGCGAG
pESC-LEU-EGFP-R	CCCAAGCTTACTTGTACAGCTCGTCCAT
pESC -LEU-EGFP-Ras2-F	GATCACTCTCGGCATGGACGAGCTGTACAAGCCGGAATTCATGCCTTTGAACAAGTCG
pESC -LEU-EGFP-Ras2-R	AGCTAGCCGCGGTACCAAGCTTTTAACTTATAATACAACA
pCDNA3.1( +)-mCherry-N-F	AATCTGTATCGCGGTCGCCGAGGCCATGGTGAGCAAGGGCGAGGA
pCDNA3.1( +)-mCherry-KDLE-R	TTTAAACGGGCCCTCTAGAGCTACAGCTCATCCTTCTTGTACAGCTCGTCCATGC
pCDNA3.1( +)-signal-BamHI-F	CCGGGATCCATGGGCGTGAAAGTGCTGTTCGCATTAATCTGTATCGCGGTCGCCGAGGCC
pEGFP-C1-LI0758-F	CAGATCTCGAGCTATGAAAAATCATACACCT
pEGFP-C1-LI0758-R	GGTGGATCCTTAACCTAAAATAGGGAAAAG
pEGFP-N1-LI0758-F	CCGGATCTCGAGATGAAAAATCATACACCT
pEGFP-N1-LI0758-R	CCGCAGAATTCGACCTAAAATAGGGAAAAG

The human embryonic kidney cell line (HEK) 293 T (Thermo Fisher Scientific, San Jose, CA, USA) was cultured in Dulbecco’s modified Eagle’s medium (DMEM, Invitrogen Tech, Carlsbad, CA, USA) supplemented with 10% (v/v) fetal bovine serum (FBS, Gibco, Waltham, MA, USA), 1 mmol L^−1^ glutamine, and 100 U mL^−1^ penicillin and streptomycin.

### Plasmid construction

The primers utilized for plasmid construction and plasmids used in this study are listed in Tables 2,  3, respectively. The LI0758 gene was amplified from genomic DNA extracted from *L. intracellularis*-positive porcine ileal mucosa and then inserted into the galactose-inducible yeast expression vector pRS416-GAL1, along with an N-terminal 3 × Flag epitope tag fragment. The DNA fragment encoding mCherry, amplified from the pBAD24-mCherry plasmid was cloned into the pRS416-GAL1 vector. Furthermore, the DNA fragments encoding full-length ScRce1 and LI0758 were separately subcloned into the pRS416-mCherry plasmid. The DNA fragment encoding full-length ScRAS2, amplified from genomic DNA extracted from the W303-1A, and EGFP, amplified from the pEGFP-C1 vector, were sequentially cloned into the pESC-Leu plasmid and fused for expression. Yeast transformation was carried out using the lithium acetate method and yeast whole cell lysates were prepared from yeast cells grown to log phase in selective medium and resolved by SDS-PAGE.

**Table 3:** **Plasmids construction in this study**

Plasmids	Genotype/Description	Source
pRS416-GAL1-ScRce1	Gal1 promoter, 3 × flag-ScRce1, URA3, ampicillin	This study
pRS416-GAL1-LI0758	Gal1 promoter, 3 × flag-LI0758, URA3, ampicillin	This study
pYES2/NTA-RipI	Gal1 promoter, EGFP-RipI, URA3, ampicillin	Lu Lab
pGBKT7	T7 promoter, c-Myc, kanamycin	Clontech
pESC-Leu	Gal1 promoter, c-Myc, ampicillin	Agilent
pESC-Leu-EGFP-Ras2	Gal1 promoter, c-Myc-EGFP-Ras2, ampicillin	This study
pBAD24-mCherry	Arabinose Bad promoter, mCherry, ampicillin	Feng shao lab
pRS416-mCherry-ScRce1	Arabinose Bad promoter, mCherry-ScRce1, URA3, ampicillin	This study
pRS416-mCherry-LI0758	Arabinose Bad promoter, mCherry-LI0758, URA3, ampicillin	This study
pCDNA3.1( +)-mCherry-KDEL	CMV promoter, signal- mCherry-KDEL, ampicillin	This study
pEGFP-C1	CMV promoter, EGFP, kanamycin	Clontech
pEGFP-C1-LI0758	CMV promoter, EGFP-LI0758, kanamycin	This study
pEGFP-N1-LI0758	CMV promoter, LI0758-EGFP, kanamycin	This study

Additionally, the DNA fragment encoding full-length LI0758 was independently subcloned into both pEGFP-C1 and pEGFP-N1 vectors. The DNA fragment, which encodes mCherry fused with a secretion signal peptide from Gaussia luciferase at its N-terminus and a C-terminal KDEL amino acid sequence, was successfully subcloned into the pcDNA3.1 vector. Subsequently, plasmid DNA was harvested from *E. coli* using an endotoxin-free DNA isolation kit (Qiagen, Valencia, CA, USA).

### Yersinia T3S secretion assay

The T3S-proficient strains *Y. pseudotuberculosis* IP2666ΔyopHOPEMT and *Y. enterocolitica* MRS40ΔyopHOPEM, along with the T3SS-null strain *Y. enterocolitica* MRS40ΔyscF, were utilized according to previously described methods [9]. Briefly, Yersinia cultures were grown in BHI medium supplemented with either 5 mM EGTA and 20 mM MgCl_2_ to induce a Ca^2+^-depleted environment (–Ca^2+^) or 5 mM CaCl_2_ to ensure Ca^2+^ availability (+ Ca^2+^). To induce gene expression, 0.2% L-arabinose was added to the cultures. Bacterial pellets and culture supernatants were separated through centrifugation, and the proteins contained within were analyzed using SDS-PAGE. Subsequently, the proteins were transferred onto PVDF membranes and probed with a rat monoclonal anti-HA antibody (CST). For immunoblot detection, secondary antibodies specific to rabbit antibodies, conjugated to horseradish peroxidase (Thermo Fisher), were employed along with ECL chemiluminescent substrate (Proteintech, Wuhan, China).

### Yeast growth assays and immunoblot analysis

Yeast growth assays and immunoblot analysis were conducted following the previously described method [21]. In brief, overnight cultures of recombinant strains grown in SD-Ura medium were washed, and their absorbance values were adjusted to OD_600_ of 1.0. Each strain was serially diluted tenfold four times and then spotted (4 µL) onto solid selective medium containing either repression (2% glucose) or induction (2% galactose). Following this, the yeast cultures were incubated at 30 °C for 2 to 4 days.

For immunoblotting, the yeast cells were harvested by centrifugation and immediately snap-frozen. Proteins were subsequently extracted from the yeast cells and subjected to SDS-PAGE analysis. The resulting gels were transferred onto PVDF membranes and probed with mouse monoclonal anti-Flag antibodies (Proteintech). The immunoblot detection was carried out using secondary antibodies specific to mouse antibodies and conjugated to horseradish peroxidase (Thermo Fisher), together with ECL chemiluminescent substrate (Proteintech).

### Yeast MAPK phosphorylation assays

Yeast cells harboring either the pRS416-GAL1-Flag-LI0758 plasmid or the control empty pRS416-GAL1-Flag vector were grown overnight in selective media supplemented with 2% raffinose. The next morning, the cultures were diluted to an OD_600_ of 1.0 in fresh media and incubated at 30 ℃ for a 2 h. Subsequently, the expression of LI0758 was then induced by the addition of 2% galactose. The cells were then incubated for another 2 h at 30 ℃ prior to the application of stress conditions, which were executed as previously detailed. Heat shock stress was administered by diluting the cells 1:1 with medium that had been preheated to 55 ℃, subsequently incubating them at 39 ℃ for 30 min. To terminate the stress response, the cells were further diluted 1:1 with an ice-cold stop mix. For the activation of the mating and invasive growth MAPK pathways, 200 nM a-factor was introduced for 15 min. Meanwhile, the HOG pathway was stimulated by the addition of 400 mM NaCl for 5 min. In every case, upon the completion of the stress protocol, the yeast cells were collected through centrifugation and promptly snap-frozen. Proteins were then extracted from the yeast cells and analyzed via SDS-PAGE. The resultant gels were transferred onto PVDF membranes and probed with specific antibodies. The phospho-p42/44 antibody was employed to detect phosphorylated Slt2, Fus3, and Kss1 in yeast, whereas the phospho-p38 antibody was used to recognize phosphorylated Hog1.

### Extract preparation and immunoblot analysis in mammalian cells

Mammalian cell extract preparation and immunoblot analysis were conducted as previously described [10]. Briefly, asynchronously growing HEK293T cells were seeded at a density of 2.5 × 10^5^ cells per well in a 6-well tissue culture plate and serum-starved overnight. Subsequently, two micrograms of plasmid DNA were transfected into the HEK293T cells using Lipofectamine^®^ 2000 reagent (Invitrogen), following the manufacturer’s protocol. After 48 h of transfection, the cells were washed with ice-cold PBS containing 1 mM Na_3_VO_4_ and 10 mM NaF, and then lysed with 300 µL of RIPA buffer containing protease inhibitor mixture (Roche Molecular Biochemicals). The protein extracted from the cells was quantified using a BCA reagent. Equal volumes of samples were subjected to SDS-PAGE, and the proteins were transferred to PVDF membranes (Immobilon-P, Merck Millipore). These membranes were then probed with mouse monoclonal anti-GFP antibodies (Proteintech).

For MAPK activation immunoblotting, protein extracts were probed with a 1:2000 dilution of primary antibodies, including rat monoclonal phospho-p38 antibody, p38 antibody, phospho-ERK42/44 antibody, ERK42/44 antibody, phospho-JNK antibody, JNK antibody (CST), and mouse monoclonal tubulin antibody (TransGen Biotech) in TBST overnight at 4 °C. This was followed by incubation with a 1:2000 dilution of either goat antimouse IgG HRP conjugate (Transgen Biotech) or goat anti-rabbit IgG HRP conjugate (Proteintech) secondary antibody for 1 h. The protein bands were visualized using ECL chemiluminescent substrate (Proteintech).

### NF-κB assay

The NF-κB assay was conducted as previously described [10]. Briefly, 5 × 10^6^ HEK293T cells were transfected with 2 μg plasmid with Lipofectamine^®^ 2000 reagent (Invitrogen). After 48 h, the cells were stimulated with TNFα (50 ng mL^−1^) for 30 min. Nuclear and cytosolic protein extracts were then prepared using the NE-PER nuclear and cytoplasmic extraction reagents (Thermo Fisher). The data were analyzed by western blotting for nuclear p65. Histone H3 and β-tubulin were used to normalize the protein concentrations of the nuclear and cytoplasmic fractions, respectively.

### Fluorescence microscopy to determine subcellular localization in mammalian cells

HEK293T cells were seeded at a density of 2.5 × 10^5^ cells per well in a 6-well tissue culture plate. The plasmids were transfected into the HEK293T cells using Lipofectamine™ 2000 (Invitrogen) according to the manufacturer’s protocol, and the plates were incubated at 37 °C for 24 h. Subsequently, the transfected cells were grown on coverslips and fixed in 4% paraformaldehyde (PFA, Sigma) in 1 × PBS for 20 min at room temperature. The cells were then rinsed in 1 × PBS before incubation in blocking solution (0.1% BSA in PBS, pH 7.4) containing 0.1% Triton for 10 min. The cells were then stained with rhodamine-conjugated phalloidin overnight at 4 °C. After rinsing 5 times in 1 × PBS, the cells were mounted on glass coverslips using DAPI-containing mounting medium (Invitrogen). The samples were visualized using conventional laser excitation and filter sets on a confocal laser scanning microscope (Nikon A1R).

### Yeast mating assays

Quantitative mating assays were performed with minor adjustments to a previous described method [22]. Briefly, MATa yeast strains (W303-1A wild-type or rce1Δste24Δ mutant, both carrying pRS416 empty vector, pRS416-ScRce1 or pRS416- LI0758 plasmid) were cultivated in SC-Ura medium for 36 h. Concurrently, MATα yeast strain W303-1B (containing pESC-LEU empty plasmid) was grown in SC-Leu medium. After standardizing cultures to OD_600_ ~ 1.0, MATa yeast suspensions were diluted ten-fold in triplicate using SC-Ura medium. Aliquots were mixed with undiluted MATα yeast and incubated in YPD medium at 30 °C for 6 h to allow mating. Yeast cells were then collected, resuspended, and plated on mating-selective (SC-Leu-Ura) and control (SC-Ura) agar plates. After 3 days of incubation, colonies were counted, adjusted for dilution, and normalized. Mating efficiencies were calculated as percentages relative to the wild-type strain.

### Fluorescence microscopy to determine subcellular localization in yeast

The plasmid pRS416-mCherry-ScRce1 or pRS416-mCherry-LI0758, along with the plasmid pESC-LEU-EGFP-RAS2, was transformed into yeast strain W303-1A. The transformed yeast was then grown in SC-URA-LEU medium at 30 °C until it reached the exponential phase (OD_600_ ~ 0.8–1.0). Afterward, the cells were pelleted, washed, and cultured in selective induction medium consisting of 2% galactose and 1% raffinose. Following 12 h of induction, the cells were fixed in 3.7% formaldehyde for 30 min at room temperature and washed twice with PBS containing 1 mg mL^−1^ BSA. The mCherry and GFP signals were visualized using conventional laser excitation and filter sets on a confocal laser scanning microscope (Nikon A1R).

## Results

### Secretion and export of LI0758 through the Yersinia T3SS system

**Figure 1:**
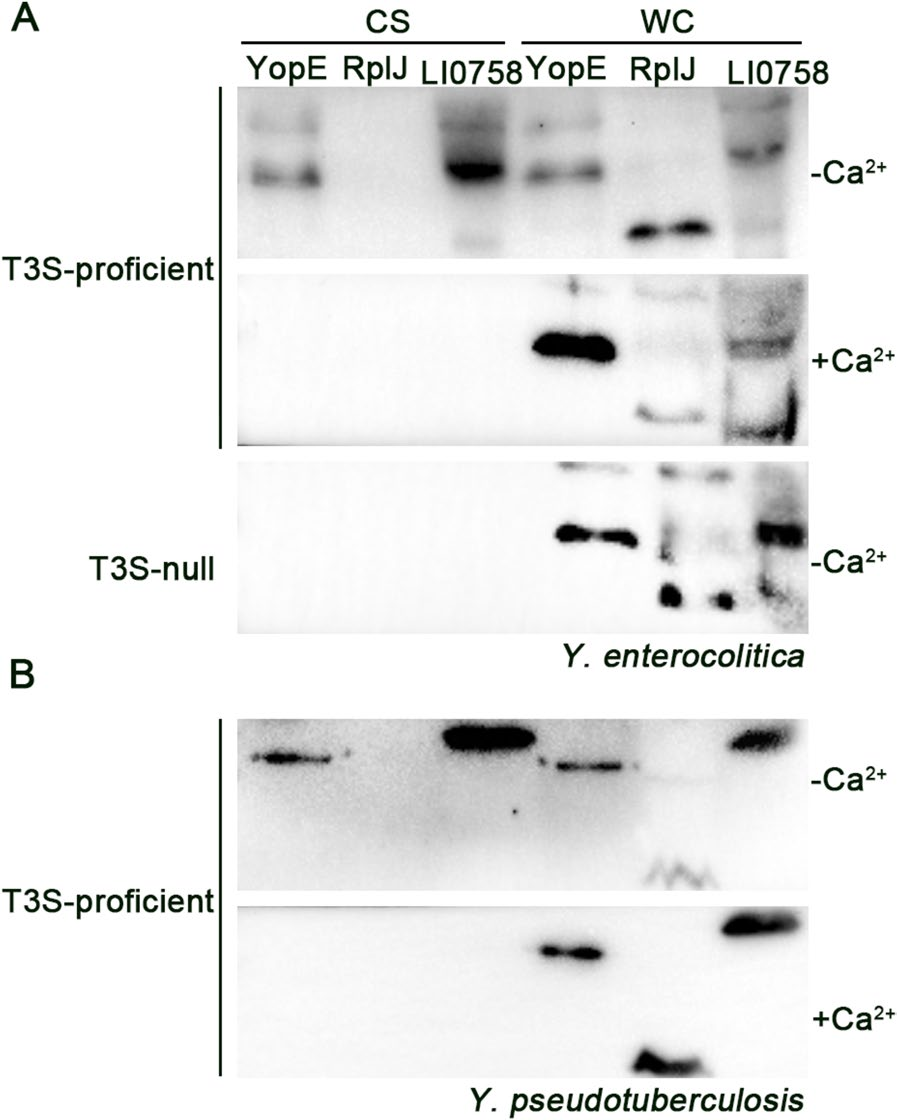
**LI0758 is a substrate of the Yersinia T3SS. A**. *Y. enterocolitica* T3S assays. Two *Y. enterocolitica* strains (T3S-proficient MRS40ΔyopHOPEM and T3S-null MRS40∆yscF), expressing HA-tagged proteins, were cultivated in BHI medium under conditions that either repressed (+ Ca^2+^) or induced (-Ca^2+^) the T3SS. After growth at 37 ℃ for 6 h with L-arabinose induction, cell-free culture supernatants (CS) and whole-cell pellets (WC) were isolated, and resolved on 12% (w/v) polyacrylamide gels. Proteins were detected by immunoblotting. **B**. *Y. pseudotuberculosis* T3S assays. The *Y. pseudotuberculosis* IP2666ΔyopHOPEMT, also expressing HA-tagged proteins, underwent the same cultivation, induction, isolation, resolution, and immunoblotting procedure as described for (**A**).

Since the T3SS apparatus structure is conserved across various pathogens, we aimed to determine whether LI0758 functions as a T3SS effector using *Y. enterocolitica* as a heterologous system. We expressed HA-tagged LI0758 in both T3S-competent (WT) and T3S-null (ΔyscF) strains of *Y. enterocolitica* under the control of an arabinose-inducible promoter. Our results showed that LI0758 was abundant in the supernatants of wild-type *Y. enterocolitica* under T3SS-inducing conditions (−Ca^2+^), but not under T3SS-repressive conditions (+ Ca^2+^). Comparable results were observed for the positive control YopE, an endogenous T3SS substrate. Importantly, these results were not due to bacterial lysis, as evidenced by the detection of the Yersinia cytoplasmic protein RplJ (a negative control) only in whole-cell pellets and not in the supernatant (Figure 1A). Furthermore, LI0758 and YopE were undetected in the supernatant of the ΔyscF strain, confirming that LI0758 secretion is dependent on a functional virulence-associated T3SS in Yersinia (Figure 1A). Consistent results were also obtained with the T3S-competent *Y. pseudotuberculosis* (Figure 1B). Overall, these findings demonstrate that LI0758 can be exported by the Yersinia T3SS.

### Growth inhibition of yeast by LI0758

**Figure 2:**
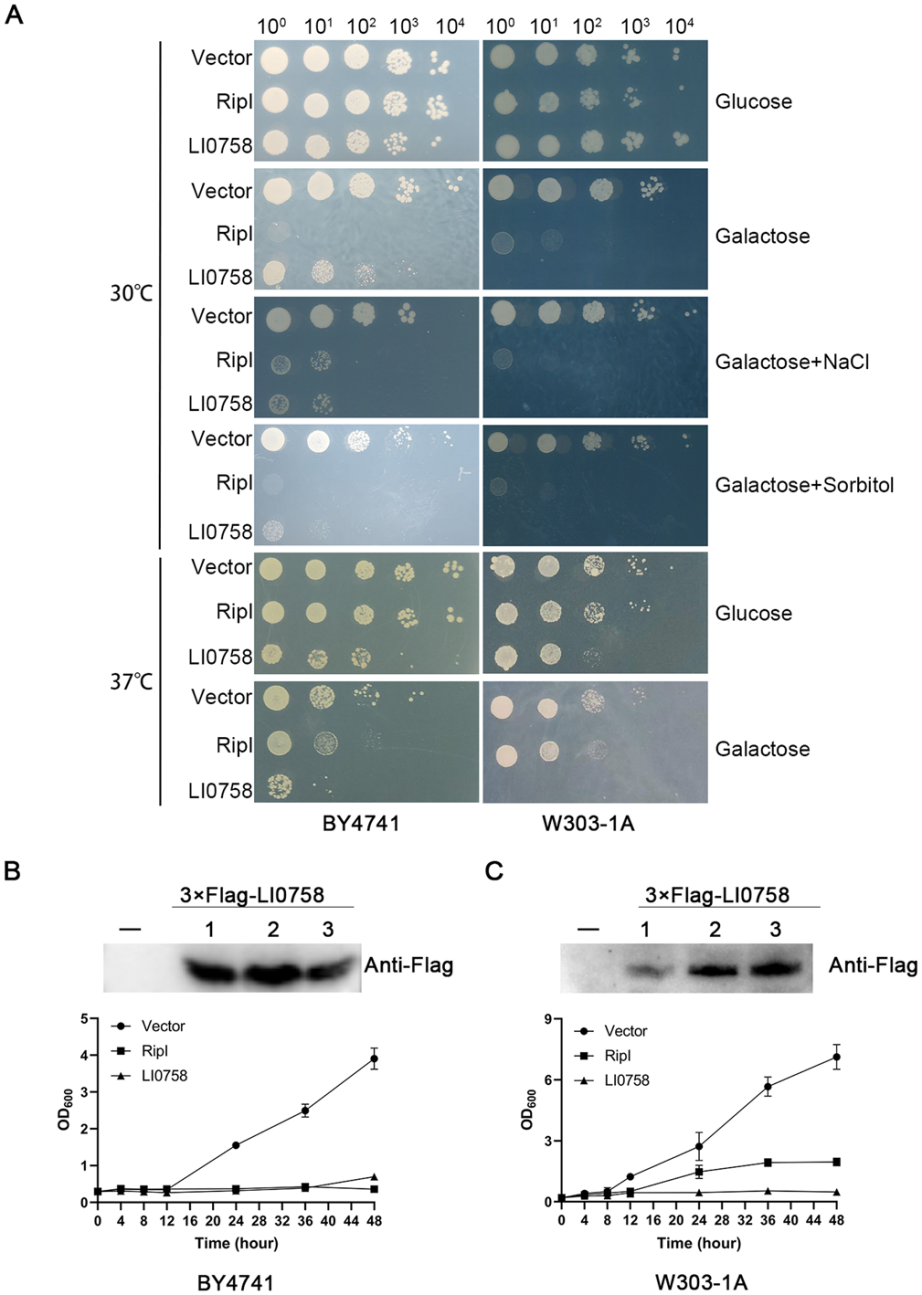
**Effect of LI0758 on yeast growth. A**. Examples of growth defects in yeast strains expressing LI0758 proteins. The yeast strains BY4741 and W303-1A, each transformed with an empty vector, RipI (serving as a positive control), and LI0758 under the regulation of the GAL1 promoter, were cultivated to late logarithmic phase in medium containing 2% dextrose. Subsequently, tenfold serial dilutions (ranging from 10^0^ to 10^4^) were spotted onto agar plates containing either glucose (as a repressor) or galactose (as an inducer), supplemented with 0.5 M NaCl or 0.8 M sorbitol. The plates were then incubated at temperatures of 30 °C or 37 °C. The experiments were conducted in triplicate, with consistent results obtained. **B**. Liquid growth assay and western blot of yeast strains BY4741. Three clones transformed with LI0758 (denoted as clones 1, 2, and 3) were randomly selected. These clones were first subjected to expression level analysis via western blotting. Subsequently, their growth over time was analyzed in a liquid growth assay. The error bars represent the standard deviation of the mean values from three independent experiments. **C**. Liquid growth assay and western blot of yeast strains W303-1A. Three clones transformed with LI0758 (denoted as clones 1, 2, and 3) were randomly selected. These clones were first subjected to expression level analysis via western blotting. Subsequently, their growth over time was analyzed in a liquid growth assay. The error bars represent the standard deviation of the mean values from three independent experiments.

The budding yeast *S. cerevisiae* is a crucial heterologous system for functionally characterizing T3SS effectors within a eukaryotic environment [23]. To explore LI0758 function, we cloned the LI0758 gene fragment under the control of the GAL1 promoter into the low copy number vector pRS416-GAL1, creating the recombinant plasmid pRS416-GAL1-Flag-LI0758. Flag-tagged LI0758 was induced for expression in the *S. cerevisiae* strain BY4741. Under inducing conditions on solid agar media, LI0758 exhibited moderate growth inhibition in yeast, although this effect was less prominent compared to the positive control RipI (Figure 2A). To further investigate the impact of LI0758 on yeast growth under stress, we introduced the gene into yeast cells and subjected them to various stressors: sorbitol, an osmotic stressor particularly toxic to HOG MAPK pathway mutants; NaCl, which imposes both osmotic and ionic stress and is detrimental to both HOG MAPK pathway mutants and those with ion homeostasis defects; and heat stress at 37 °C, which causes a multitude of effects in yeast [24]. We observed that all three stressors augmented the growth inhibition caused by LI0758. Notably, even under non-inducing conditions and at 37 °C, the basal expression of LI0758 had a mild inhibitory effect on yeast growth. Upon induction, LI0758 severely inhibited yeast growth (Figure 2A).

To verify LI0758 function in another genetic background, we transformed the pRS416-GAL1-Flag-LI0758 construct into strain W303-1A, where LI0758 exhibited severe growth inhibition (Figure 2A). Growth curves in liquid medium corroborated these results (Figures 2B, C). Western blot analyses confirmed similar LI0758 expression levels in different strains (Figures 2B, C). Overall, these results demonstrate that LI0758-induced expression targets conserved signaling pathways within eukaryotic cells and inhibits *S. cerevisiae* growth. Given that the inhibitory effect of LI0758 was more pronounced in the W303-1A strain than in BY4741, W303-1A was chosen for further experiments.

### LI0758 modulate the MAPK signaling pathway in yeast

**Figure 3:**
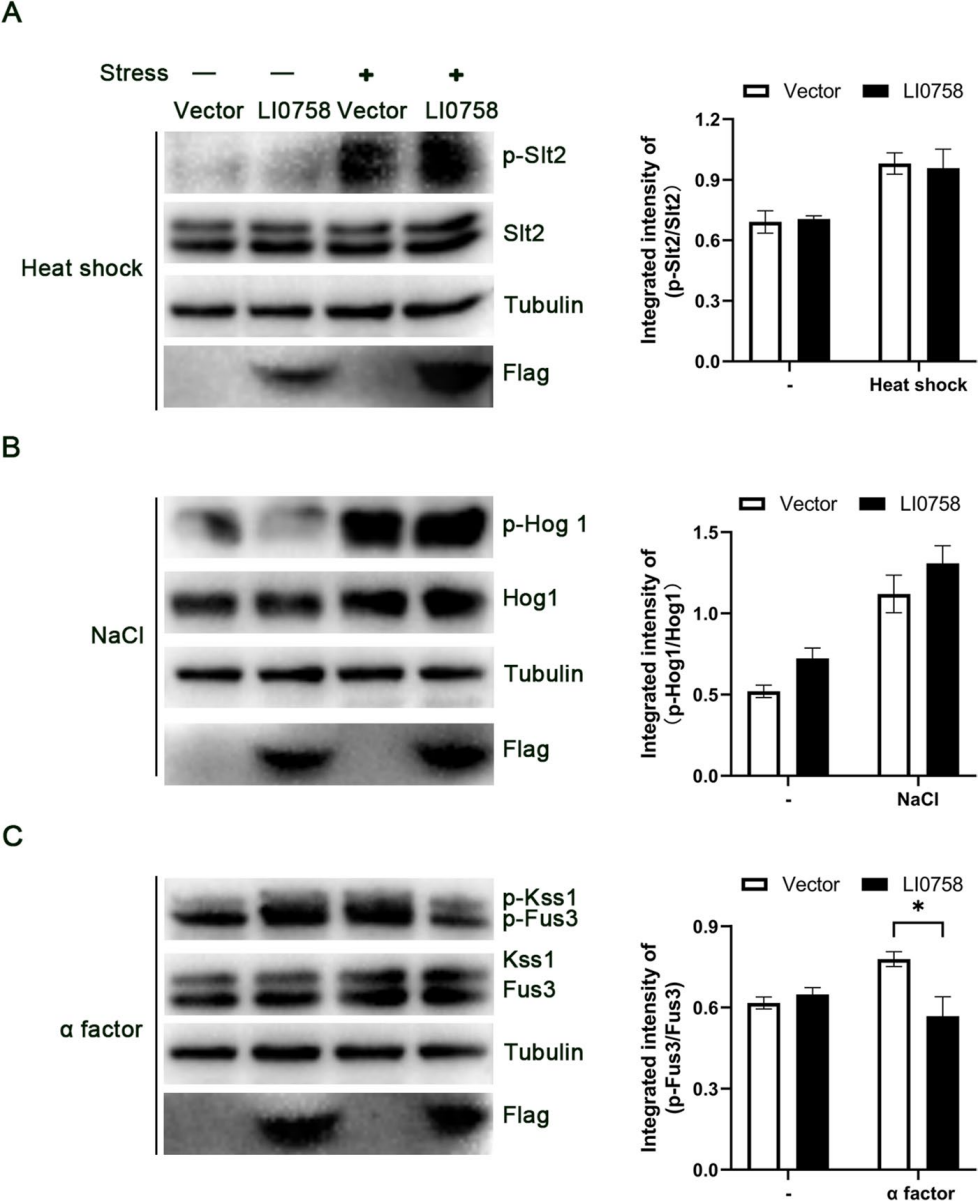
**Effect of LI0758 on yeast MAPK signaling pathway. A**. Heat shock activation of CWI (SLT2 phosphorylation) pathway. Yeast strains, transformed with control or LI0758-expressing plasmids, were heat-shocked at 39 °C for 30 min after 2 h induction. Immunoblotting showed total and activated SLT2 levels, with tubulin as a loading control. LI0758 expression was confirmed using an anti-Flag antibody. **B**. NaCl activation of HOG (HOG1 phosphorylation) pathway. Yeast strains, harboring control or LI0758-expressing plasmids, were treated with NaCl to induce a state of high osmolarity for 5 min after a 2 h induction period. Immunoblotting revealed both the total and activated forms of HOG1, with tubulin serving as a loading control. LI0758 expression was verified using an anti-Flag antibody. **C**. α-Factor activation of pheromone response (FUS3 phosphorylation) and filamentous growth (KSS1) pathways. Yeast strains, transformed with control or LI0758-expressing constructs, were exposed to 200 nM α-factor for 15 min following a 2 h induction. Immunoblotting demonstrated the total and activated states of FUS3 and KSS1, with tubulin as a loading control. The expression of LI0758 was confirmed by an anti-Flag antibody.

The severe growth inhibition observed in yeast cultured in liquid media can be attributed to LI0758’s inhibition of multiple yeast MAPK signaling pathways [25]. To further elucidate the specific action of LI0758, we utilized the cross-reactivity of mammalian phospho-specific MAPK antibodies with phosphorylated MAPK in yeast. Our findings reveal that upon induction of LI0758 expression, it slightly activates the phosphorylation of FUS3 in the pheromone response pathway and HOG1 in the high osmolarity pathway, yet it does not elicit phosphorylation of SLT2 in the cell wall integrity pathway (Figures 3A–C).

Given that MAPK pathway activation is minimal in wild-type yeast under standard laboratory conditions, we assessed LI0758’s ability to block MAPK pathway activation induced by heat stress, hypoosmotic shock, or mating factor treatment. Our results indicate that LI0758 expression does not inhibit the phosphorylation of SLT2, HOG1, and KSS1, but selectively inhibits the phosphorylation of FUS3 after pheromone treatment, without altering total Fus3 levels (Figures 3A–C). In conclusion, our experimental data suggest that the MAPK pathway is a target of LI0758. To enhance our understanding of LI0758’s effects on the MAPK pathway, we intend to conduct additional validation experiments in mammalian cells.

### Activation of MAPK and NF-κB signaling pathways by LI0758 in HEK293T cells

**Figure 4:**
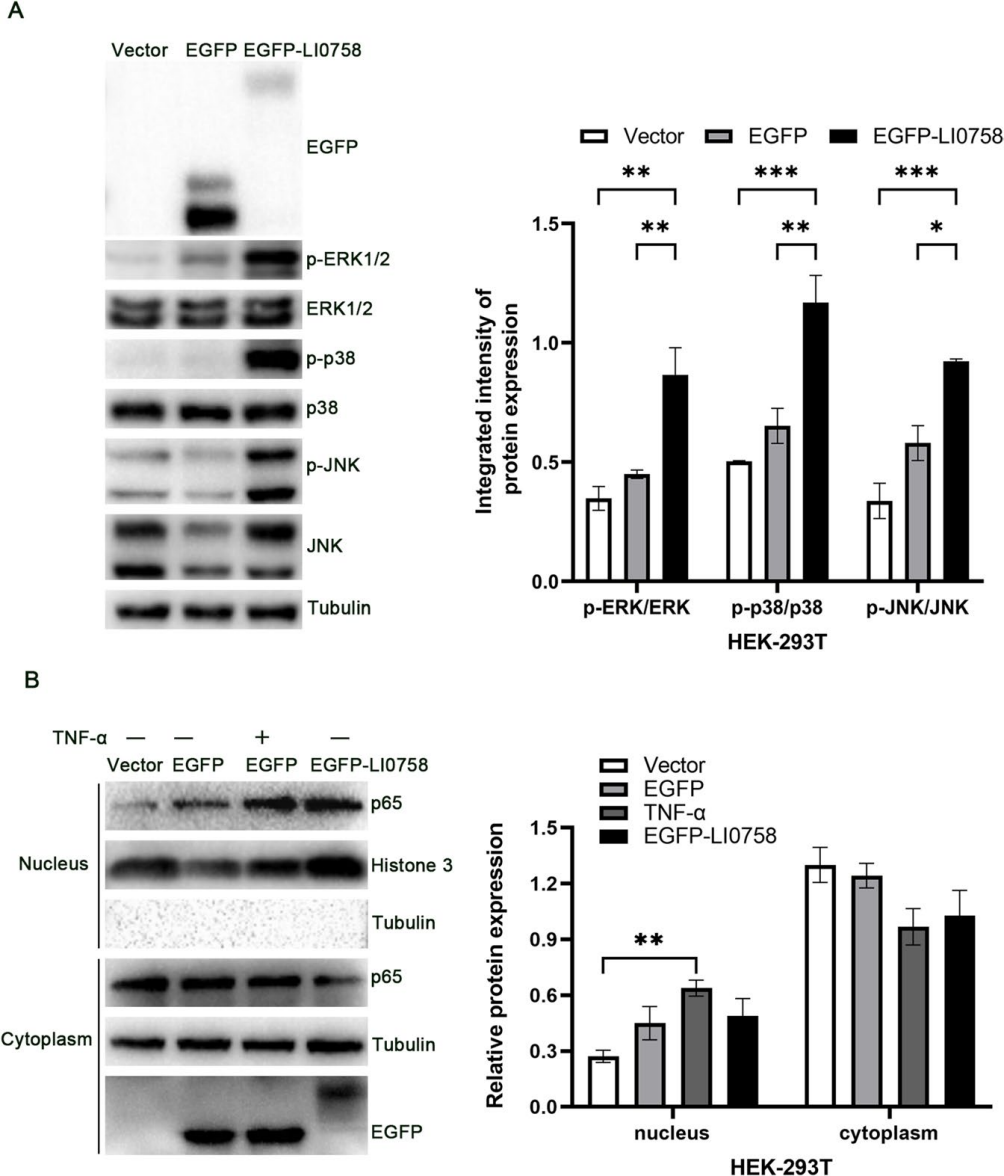
**LI0758 activates MAPK and NF-κB signaling in HEK293T cells. A**. LI0758 induces MAPK activation in HEK293T cells. Immunoblots were performed on extracts from HEK293T cells transfected with pEGFP-C1-LI0758 or pEGFP-C1 (as a negative control) for 48 h. Cell lysates were analyzed for total and phosphorylated forms of ERK1/2, p38, and JNK, along with tubulin as a loading control. Data are presented as the means ± SEM of three independent experiments. Statistical significance was determined by a two-tailed unpaired Student’s *t*-test (*p < 0.05, **p < 0.01, ***p < 0.001). **B**. LI0758 modulates NF-κB signaling in the nucleus and cytoplasm of HEK293T cells. Immunoblots show the distribution of proteins in the nucleus and cytoplasm of HEK293T cells transfected with pEGFP-C1-LI0758 or pEGFP-C1 for 24 h, with or without exposed to 10 ng mL^−1^ TNF-α for 45 min. Specific antibodies were used to detect p65 (a component of NF-κB), Histone H3 (a nuclear marker), EGFP (to confirm LI0758 expression), and tubulin (as a control). Data are presented as the means ± SEM of three independent experiments. Statistical significance was determined by a two-tailed unpaired Student’s *t*-test (*p < 0.05).

To test whether LI0758 alters MAPK signaling during transfection, we compared MAPK phosphorylation patterns in mammalian cells transfected with EGFP-LI0758 or empty vector. We transiently transfected HEK293T cells with a recombinant LI0758 expression plasmid for 36 h and examined whole cell lysates using western blot analysis. The results showed that EGFP-LI0758 expression induced significant increases in the phosphorylation of ERK1/2, p38, and JNK1/2 compared with the empty EGFP control (Figure 4A). Using an antibody against GFP, we detected that the expression level of EGFP-LI0758 was lower than that of the control, thus ruling out the possibility that differences in phosphorylation phenotypes were due to variations in expression levels (Figure 4A). These observations demonstrate that LI0758 can activate multiple downstream MAPK pathways.

Given that EGFP-LI0758 expression augments ERK1/2 phosphorylation, we explored the possible impact of LI0758 on the nuclear translocation of the NF-κB transcription factor p65. Our results revealed a modest increase in p65 nuclear abundance in cells transfected with EGFP-LI0758, albeit not as pronounced as that observed with TNFα treatment (Figure 4B). These observations indicate that transient transfection of LI0758 into mammalian cells leads to a slight activation of the NF-κB pathway.

### Characterization of *L. intracellularis* LI0758

**Figure 5:**
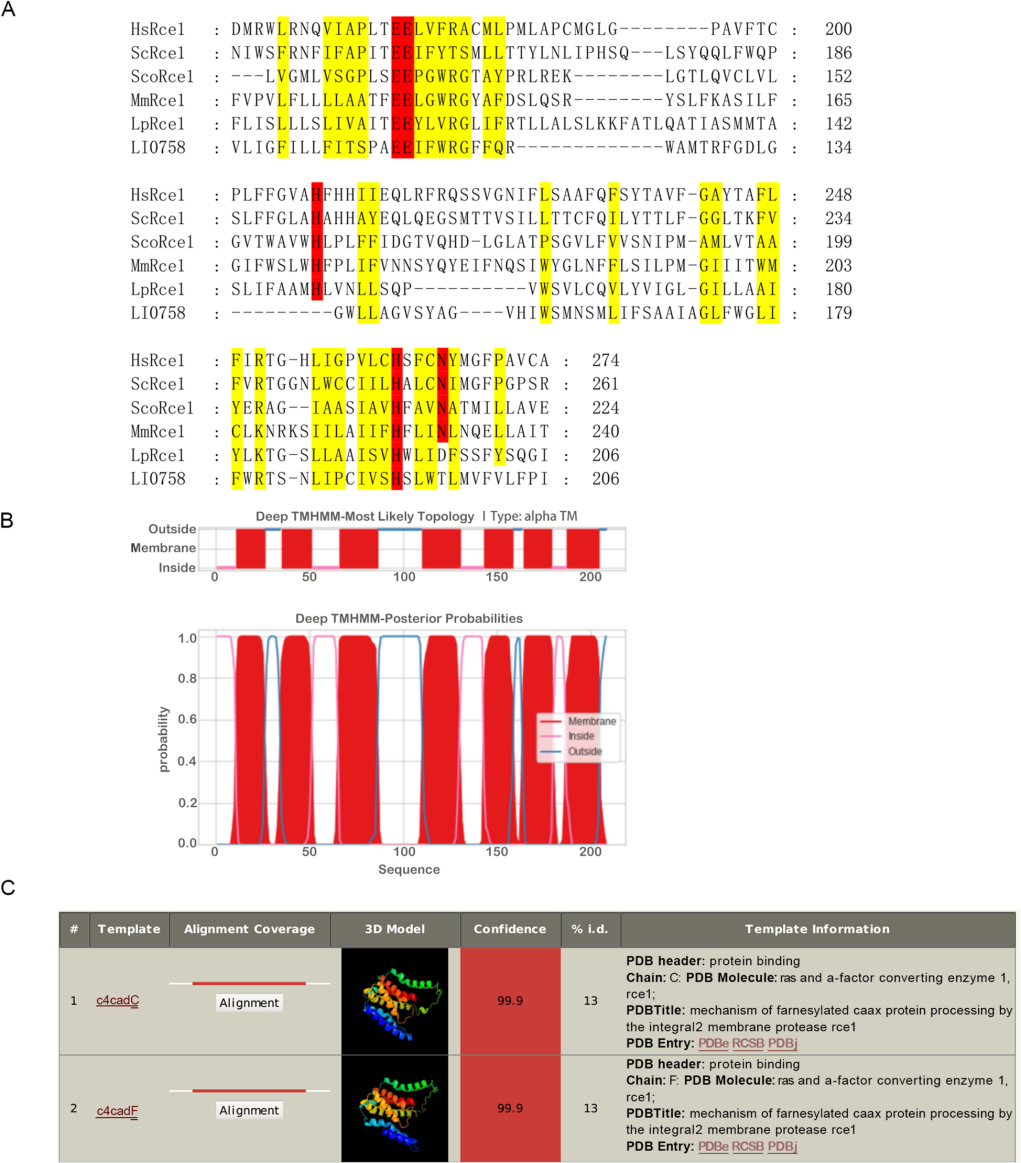
**Characterization of LI0758. A**. Multiple sequence alignment of ABI domains from Rce1 homologues across all three domains of life. HsRce1: *Homo sapiens* (UniProt: Q9Y256), ScRce1: *S. cerevisiae* (UniProt: Q03530), ScoRce1: *Streptomyces coelicolor* (UniProt: Q9XAK4), MmRce1, *Methanococcus maripaludis*, (UniProt: Q6LZY8), LpRce1: *Lactobacillus plantarum*, (UniProt: C6VK86) and LI0758: *L. intracellularis*, (UniProt: Q1MQB5). Critical conserved residues essential for activity are highlighted in red. **B**. Predicted transmembrane structure of LI0758 obtained using the TMHMM server. **C**. Top two protein templates, ranked by confidence, resulting from sequence alignment of LI0758 using Phyre2 software.

To gain deeper insights into the mechanisms underlying LI0758-mediated activation of MAPK and NF-κB, we undertook a comprehensive series of bioinformatics analyses. Initially, we annotated the domain architecture of LI0758’s amino acid sequence by employing the HMMSCAN tool in conjunction with the Pfam database [26]. Our analysis unveiled two notably matching domains, both belonging to the Rce1-like family. Precisely, domain A resides from positions 3 to 91 with an E-value of 0.39, whereas domain B (alternatively termed the ABI domain) extends from positions 106 to 199, exhibiting a more pronounced E-value of 1.4e-17. Buoyed by the high significance of domain B and drawing on prior research, we executed a multiple sequence alignment focusing on the C-terminal ABI domains of LI0758 and its Rce1 orthologs spanning three domains of life. Our findings revealed that both LI0758 and its Rce1 orthologs harbor several conserved amino acids, postulated to act as catalytic residues: specifically, two contiguous glutamates and two distinct histidines (Figure 5A).

Subsequently, we utilized the SignalP signal peptide analysis tool to scrutinize the first 30 amino acids at LI0758’s N-terminus [27]. The outcomes indicated an absence of a signal peptide, implying that LI0758 might not be secreted via the signal peptide pathway. This aligns with our earlier conclusion that LI0758 is secreted through a type 3 secretion system (Figure 1).

To further explore LI0758’s structural features, we leveraged the DeepTMHMM server to predict its transmembrane structure [28]. The findings suggested that LI0758 potentially possesses 6–7 transmembrane segments, classifying it as a multiple transmembrane protein (Figure 5B). This observation correlates with the hydrophobicity and multiple sequence alignment results of Rce1 orthologs derived from humans (HsRce1), *S. cerevisiae* (ScRce1), and various other species [14]. Depending on the species, Rce1 orthologs are anticipated to exhibit 7 or 8 transmembrane helices [14].

In pursuit of a more profound understanding of LI0758’s structure and function, we aligned it with known structural sequences using Phyre2.2 software [29]. We presented the top three protein templates ranked by confidence level from the sequence alignment with LI0758 (Figure 5C). These findings imply a strong homology between LI0758 and Rce1. Despite a relatively low sequence match percentage, the model’s results carry significant weight due to a high confidence level of 99.9%.

Finally, we predicted LI0758’s subcellular localization in prokaryotic and eukaryotic cells using ProtComp and Cell-PLoc 2.0, respectively [30]. The results indicated that, in line with Rce1 orthologs, LI0758 is probably situated on the bacterial plasma membrane and localized to the ER in eukaryotic cells [14].

In conclusion, the aforementioned predictive outcomes uniformly indicate that LI0758 is likely an ortholog of Rce1, the CAAX protein endoprotease found in eukaryotes.

### Localization of LI0758 at the ER in HEK293T cells

**Figure 6:**
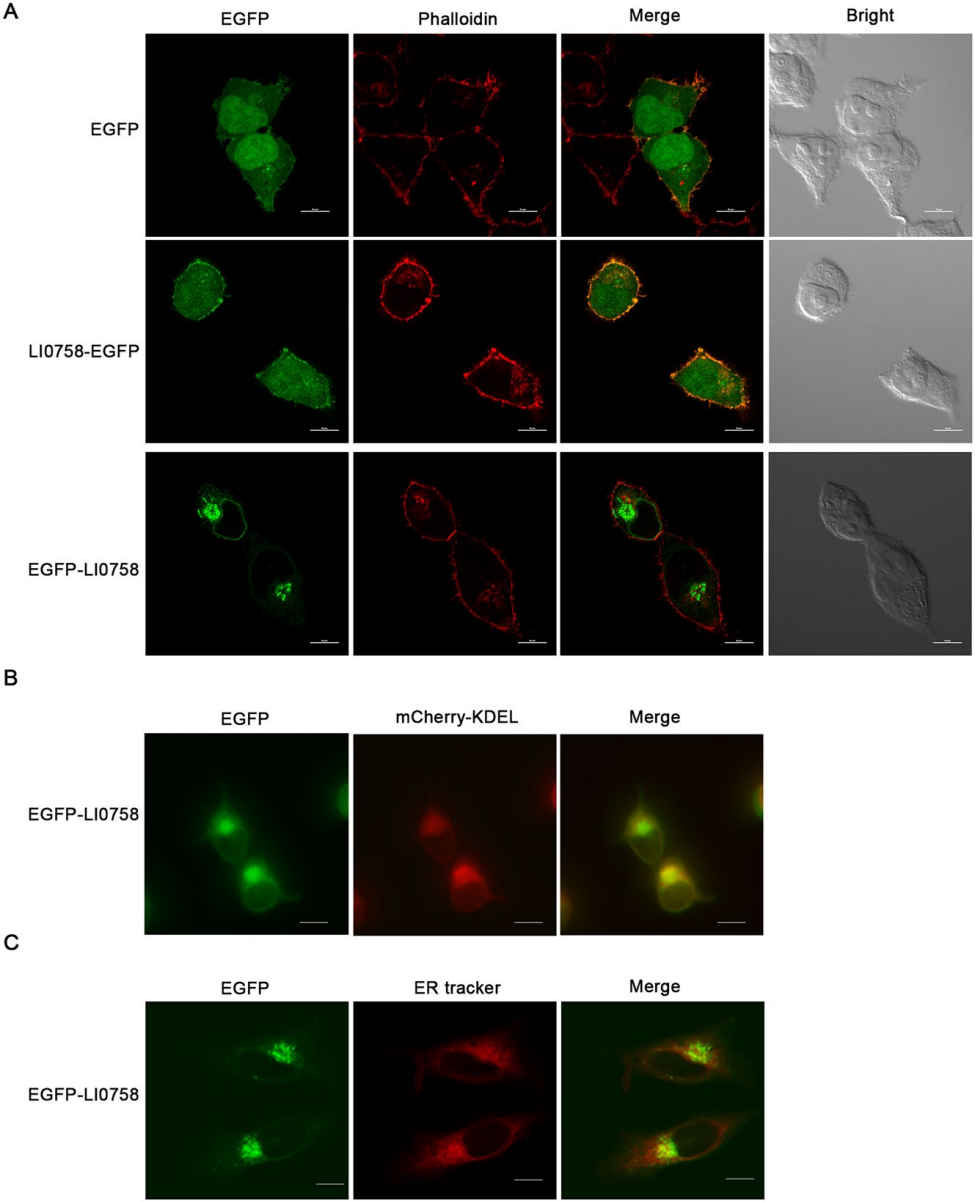
**Localization of LI0758 to the endoplasmic reticulum (ER) in HEK293T cells. A**. Transfection and actin cytoskeleton staining. HEK293T cells were individually transfected with pEGFP-C1-LI0758, pEGFP-N1-LI0758, and an empty pEGFP-C1 vector for 24 h. Subsequently, the actin cytoskeleton of these cells was stained with rhodamine-phalloidin. Visualization was achieved using a laser scanning confocal microscope, allowing for precise localization of the transfected constructs and actin cytoskeletons within the cells. **B**. Co-localization with an ER retention signal. To assess the localization patterns of the EGFP-LI0758 fusion protein, HEK293T cells were co-transfected with mCherry-KDEL (serving as an ER retention signal) and EGFP-LI0758 for 24 h. Live-cell imaging was then performed using fluorescence microscopy with the appropriate filters. **C**. ER tracker dye staining. The EGFP-LI0758 plasmid was transiently transfected into HEK293T cells for 24 h. The cells were subsequently stained with an ER tracker dye, which specifically targets and stains the ER compartments. Visualization under a fluorescence microscope allowed for the observation of the localization and distribution of the EGFP-LI0758 fusion protein. Scale bar: 10 μm.

To verify LI0758 localization characteristics, we fused the LI0758 gene with either the N-terminus or C-terminus of the EGFP gene and expressed the fusion proteins in HEK293T cells. Confocal laser scanning microscope observation showed that LI0758 fused with EGFP at the C-terminus exhibited a diffuse distribution, consistent with the empty vector (Figure 6A). Conversely, LI0758 fused with EGFP at the N-terminus displayed a specific localization pattern, forming a ring-like structure with a reticular appearance, speculated to be the ER (Figure 6A).

To further verify ER localization, we co-transfected a plasmid containing mCherry with an ER retention signal (KDEL) and EGFP-LI0758 into HEK293T cells. The results showed that mCherry-KDEL and EGFP-LI0758 exhibited similar localization patterns, with significant colocalization (Figure 6B). Additionally, ER-Tracker dye labeling confirmed LI0758 localization on the ER (Figure 6C). Overall, these results indicate that LI0758 localization is consistent with bioinformatics predictions and RCE1 localization, particularly on the ER within mammalian cells.

### In vivo activity and function of LI0758

**Figure 7:**
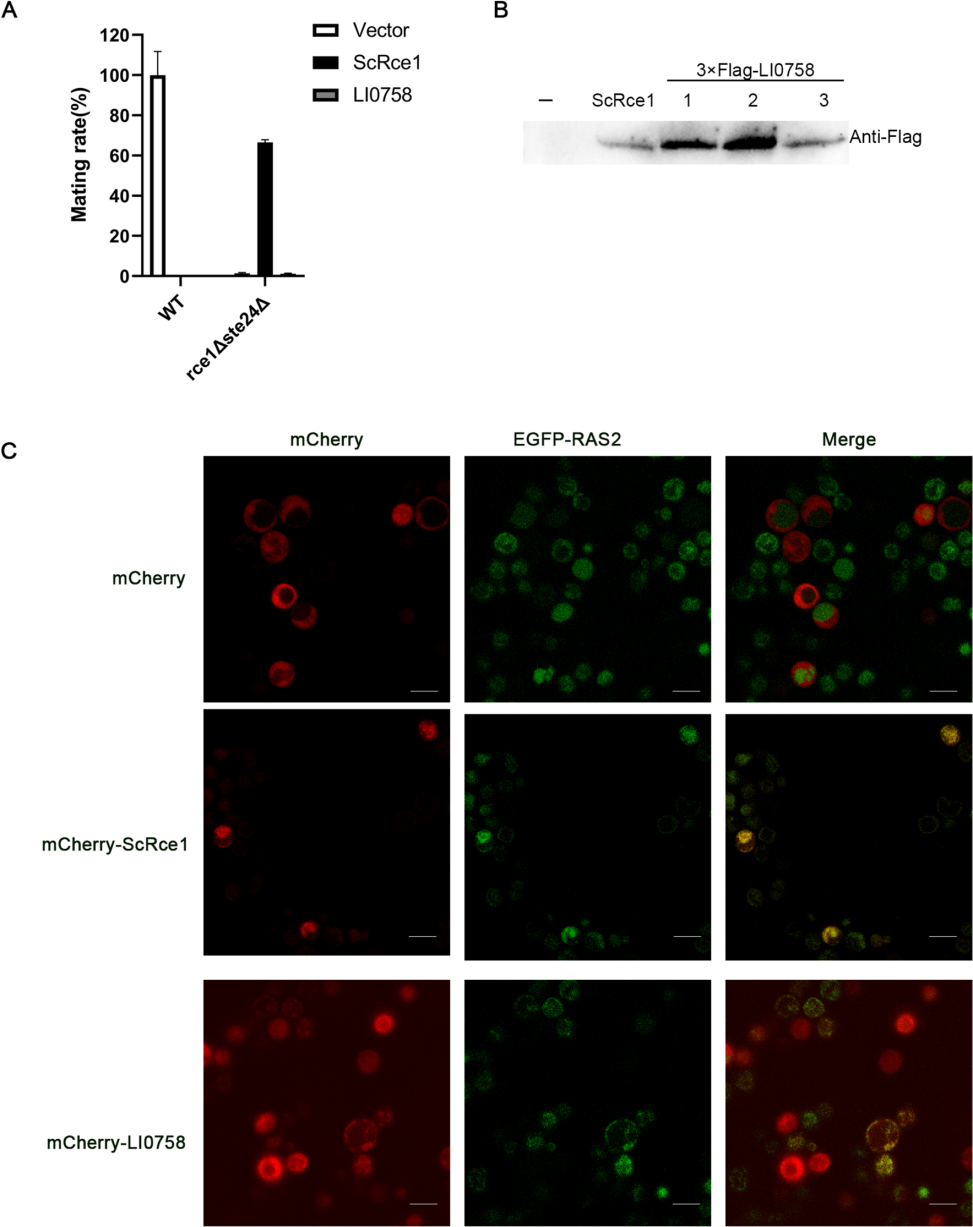
**In vivo activity and function analysis of LI0758. A**. Mating efficiency of yeast strains expressing LI0758. Quantitative mating assays were conducted in yeast as described in Materials and methods section. Each assay was conducted in triplicate, with the wild-type strain serving as the reference (set to 100%). The mating efficiency of mutant strains is expressed as a percentage relative to the wild-type. The error bars indicate the standard deviation from the mean values. **B. **Expression confirmation of ScRce1 and LI0758 by western blot. Western blot analysis was conducted using an anti-Flag antibody to verify the expression of ScRce1 and LI0758. Numbers 1, 2, and 3 indicate three randomly selected clones from yeast transformed with LI0758, which were later used in the mating experiments shown in **A**. **C**. Localization of GFP-Ras2 in rce1Δ mutant strains. To compare the localization patterns and intensities of the GFP-Ras2 fusion protein, rce1Δ mutant strains were created that expressed GFP-Ras2 along with either an mCherry empty vector, mCherry-tagged ScRce1, or mCherry-tagged LI0758, all under the control of the GAL1 promoter. These strains were grown in SC-Leu-Ura medium, diluted to a 1:10 ratio, and induced for expression over 8 h at 30 ℃. Live-cell imaging was performed using fluorescence microscopy with appropriate filter settings. The scale bar is 10 μm.

To investigate whether LI0758 exhibits in vitro proteolytic activity, we attempted to express and purify LI0758 fused with various tags, but all attempts were unsuccessful due to its multiple transmembrane properties [19]. To determine whether LI0758 possesses RCE1 proteolytic activity in vivo, we used a yeast quantitative mating assay as described by Huyer et al. [31]. We constructed null alleles of RCE1 (rce1Δ) and STE24 (ste24Δ), and a double mutant (rce1Δ ste24Δ) in the W303-1A strain background. In line with previous research, only the double mutants (rce1Δ ste24Δ) were entirely impaired in mating, whereas both single mutants (rce1Δ and ste24Δ) retained some activity [31]. Complementation with ScRce1 partially rescued the mating defect in double mutants, whereas complementation with LI0758 did not exhibit such an effect (Figure 7A). Utilizing Flag as an antibody, we confirmed that the expression level of Flag-LI0758 was comparable to that of Flag-ScRce1, thereby eliminating the possibility that differences in mating phenotypes were attributed to variations in expression levels (Figure 7B). Therefore, the yeast mating assay indicates that LI0758 does not possess similar in vivo activity to Rce1.

Apart from yeast a-factor pheromone serving as a reporter gene to monitor the in vivo activity and function of RCE1, GFP tagging of Ras and other Ras-related GTPases also constitutes an effective method for assessing the crucial role of Rce1 in regulating the subcellular localization of small GTPases [13, 14]. To further elucidate LI0758 activity, we examined the subcellular localization pattern of fluorescently tagged RAS2 protein in the yeast rce1Δ mutant strains. In the rce1Δ mutant strain with an empty vector, EGFP-RAS2 was primarily localized to the cytoplasm. However, when LI0758 was introduced, it co-localized with RAS2 and restored the plasma membrane localization of Ras, mimicking the function of ScRce1 (Figure 7C). These observations suggest that, while prenylation itself is required for RAS2 membrane localization, LI0758 restores the plasma membrane localization of Ras2 in rce1Δ mutant strains.

## Discussion

Despite decades of research on *L. intracellularis*, our current comprehension of its molecular pathogenesis remains quite limited due to its obligate intracellular nature, genetic intractability, and the lack of an in vitro mature bacterial model [2]. Furthermore, there is limited understanding regarding the T3SS effectors of this pathogen, which are facilitated by a T3SS enabling the transfer of proteins into targeted host cells to modify their functions. Our study identified LI0758, a T3SS effector secreted by Yersinia, which suppresses yeast growth and activates MAPK and NF-κB signaling pathways in mammalian cells, potentially contributing to inflammatory responses. The bioinformatics and fluorescence localization analyses have collectively revealed that LI0758 is an ortholog of Rce1, exhibiting a comparable localization pattern within the ER. Despite displaying unique activity in the yeast a-factor reporter system, LI0758 restores Ras2’s proper location in rce1Δ mutant strains, indicating a functional similarity to Rce1. These discoveries highlight LI0758’s role in activating MAPK and NF-κB signaling pathways in host cells, potentially modulating the localization and function of CAAX proteins, which may indirectly benefit bacterial survival or virulence. Continued investigation holds promise for revealing novel bacteria-host interaction mechanisms and fostering the development of novel treatments for proliferative enteritis.

In both our current and previous research endeavors, we have consistently identified the *L. intracellularis* proteins, namely LI0758, LI0666, LI1158, LI1159 and LfliC, as T3SS substrates through in vitro experiments utilizing Yersinia [9–11]. Following successful invasion of host cells, *L. intracellularis* promptly exits the membrane vesicles and proliferates within the cytoplasm, exhibiting behavior akin to Shigella. The obligate intracellular lifestyle of *L. intracellularis* presents a significant challenge in identifying and functionally characterizing its effectors. To address this, we propose employing Shigella as a heterologous expression system that specifically targets the host cells of *L. intracellularis*, namely IPEC-J2, in order to validate our earlier findings derived from transient transfections in mammalian cells. This methodology may help elucidate the underlying mechanisms of effector function and could potentially minimize interferences from functional redundancy or antagonism among different effectors, offering new perspectives on the pathogenic mechanisms of *L. intracellularis*.

In this study, we employed two yeast strains with distinct genetic backgrounds, BY4741 and W303-1A, to conduct a thorough analysis of the impact of LI0758-induced expression on the growth of different yeast strains. The results indicated that the growth inhibition elicited by LI0758 induction in BY4741 was milder compared to that in W303-1A; it was only under supplementary sorbitol stress that the growth inhibitory effects induced by LI0758 became comparable between the two strains (Figure 2). Genetic analysis revealed a 0.08% nucleotide difference rate between BY4741 and W303 strains, with multiple substantial fragment deletions in W303-1A relative to BY4741 [32]. Further exploration is needed to delve into the disparities in growth inhibition elicited by LI0758 in yeast strains with differing genetic backgrounds. Additionally, we noted that while LI0758 activates the MAPK signaling pathway in mammalian cells, it does not evoke a corresponding strong activation of this pathway in yeast. Furthermore, its expression in yeast does not obviously suppress pathway activation under stressful conditions. The severe growth inhibitory effect of LI0758 in W303-1A suggests its potential influence on conserved signaling pathways in eukaryotes, including cell cycle, cytoskeleton, and cell death. Subsequent experiments will investigate its mechanism of action through transcriptome analysis, proteomic analysis, pathogenic genetic array analysis, multicopy suppressor screening, and validation in mammalian cells.

When infected by pathogenic bacteria, hosts activate complex signaling pathways, notably the MAPK and NF-κB pathways, which then stimulate the transcription of downstream inflammatory factors [5]. T3SS effectors often intervene in these two crucial pathways, disrupting the host’s inflammatory response [5]. Our laboratory has found that *L. intracellularis*’ T3SS effector LI1035 inhibits MAPK phosphorylation, whereas LfliC and LI0758 promote it and activate the NF-κB pathway [10, 21]. In our future work, we aim to discover more T3SS effectors in *L. intracellularis* that target these signaling pathways. Reports indicate that enteropathogenic bacteria secrete various effectors that specifically affect the host’s MAPK and NF-κB pathways, either activating or inhibiting them [5]. Therefore, when analyzing each stage of the infection process, it’s crucial to consider the significant impact of *L. intracellularis* effectors on host responses. These proteins may act synergistically or independently on these two key pathways, employing diverse mechanisms and following a specific spatiotemporal sequence.

Our study utilized various bioinformatics analyses and fluorescence co-localization techniques to demonstrate that LI0758, as an Rce1 ortholog, exhibits similar localization patterns on the ER and can restore membrane localization of yeast Ras2 in the rce1Δ mutant. Our results indicate that the transient expression of EGFP-LI0758 markedly activates the MAPK pathway in mammalian cells. Therefore, it is essential to delve deeper into whether LI0758 activates the MAPK pathway by altering Ras’s plasma membrane localization in these cells. This requires effectively knocking down endogenous Rce1 using siRNA technology or conducting thorough analyses of Rce1-deficient cells [33]. Notably, LI0758 does not rescue the mating defect of double mutants in quantitative mating assays, unlike other known RCE1 proteins and their orthologs, emphasizing its selective action on CAAX proteins. Given the abundance of CAAX proteins in eukaryotes, which play crucial roles in cell signaling and regulatory processes like cell proliferation, differentiation, metabolism, and apoptosis, a key future research direction will be to investigate which CAAX proteins, apart from RAS, LI0758 may target to promote bacterial proliferation within host cells.

Through multiple sequence alignment analysis, we have revealed that LI0758 and its direct Rce1 orthologs contain several conserved amino acids, which are presumed to be catalytically functional residues (Figure 5). However, our current study has not included mutation experiments targeting these conserved sites to fully explore the influence of pivotal amino acids on LI0758’s activity and function. Moving forward, we are keen to systematically undertake these experiments, aiming to decipher the precise roles of key amino acids in LI0758’s function and, consequently, provide profound understanding of its contribution to bacterial infections.

In conclusion, despite the absence of prenylation modification in prokaryotes, the eukaryotic RCE1 enzyme, crucial for this process, boasts numerous orthologs spanning all three domains of life [14]. Its evolutionary mechanism and significance merit deeper investigation. Specifically, examining the function of LI0758, an RCE1 homolog from *L. intracellularis* and a T3SS effector, is anticipated to reveal its unique impact on host prenylation modification and the MAPK pathway, thereby shedding light on LI0758’s critical role in bacterial infection and proliferation. This exploration not only promises to uncover new bacteria-host interaction patterns but also to lay the groundwork for developing novel therapeutics against proliferative enteritis.
